# ENHANCING ACCESS AND QUALITY OF RURAL VETERANS’ CARE THROUGH TELEMEDICINE IMPLEMENTATION

**DOI:** 10.1093/geroni/igz038.2262

**Published:** 2019-11-08

**Authors:** Aaron T Seaman, Samantha Solimeo, Byron Bair

**Affiliations:** 1 Iowa City VA Healthcare System, Iowa City, Iowa, United States; 2 Center for Access and Delivery Research and Evaluation, Primary Care Analytics Team, Iowa City VA Health Care System, Iowa City, Iowa, United States; 3 VA Salt Lake City Healthcare System, Salt Lake City, Utah, United States

## Abstract

Telemedicine, a promising approach for clinicians to provide care to patients who are unable to attend face-to-face encounters, has been embraced by the Veterans Health Administration to improve the delivery of specialty care to rural Veterans and their caregivers. Presenters in this symposium report on the potential and challenges of telemedicine in a variety of specialty care contexts. Stakeholder (Veteran, caregiver, and provider) perspectives are discussed. Hung et al.’s study examines the implementation of a teleconsultation intervention designed to connect geriatric teams with rural clinics, reporting on both patient outcomes and the challenges of implementation variability that teams encountered. Solimeo, et al.’s qualitative study of primary care providers’ experience of an osteoporosis telemedicine clinic’s outreach and care delivery demonstrates stakeholder acceptance as well as potential missed opportunities for patient and provider education. Findings from Nichols and Martindale-Adams’ mixed methods evaluation of a telephone intervention to assist caregivers of older, rural-dwelling Veterans demonstrate the impact the intervention has had on participants’ perceptions of and abilities to care for their family members and themselves. Hicken et al. present on the implementation of a videoconferencing intervention to provide in-home support to rural Veterans and their caregivers, reporting on both provider and patient/caregiver experiences of the intervention and its implementation. The four studies highlight the unique ways telemedicine can improve care and the necessity of including stakeholder perspectives across the implementation process.

